# Applications of genetic code expansion and photosensitive UAAs in studying membrane proteins

**DOI:** 10.1515/biol-2022-0752

**Published:** 2023-10-10

**Authors:** Shu Zhao, Dong Liu

**Affiliations:** School of Life Sciences, Nantong Laboratory of Development and Diseases, Nantong University, Nantong, 226019, China

**Keywords:** genetic code expansion, photosensitive UAAs, light control, ion channels, membrane receptors

## Abstract

Membrane proteins are the targets for most drugs and play essential roles in many life activities in organisms. In recent years, unnatural amino acids (UAAs) encoded by genetic code expansion (GCE) technology have been widely used, which endow proteins with different biochemical properties. A class of photosensitive UAAs has been widely used to study protein structure and function. Combined with photochemical control with high temporal and spatial resolution, these UAAs have shown broad applicability to solve the problems of natural ion channels and receptor biology. This review will focus on several application examples of light-controlled methods to integrate GCE technology to study membrane protein function in recent years. We will summarize the typical research methods utilizing some photosensitive UAAs to provide common strategies and further new ideas for studying protein function and advancing biological processes.

## 1 Introduction

Membrane proteins play significant roles in cell life activities such as cell proliferation, cell differentiation, signal transmission, and material transport. They are the main targets of drugs and most therapeutic drugs achieve therapeutic effects by interacting with the membrane proteins. There have been many studies on drug targets for membrane proteins, such as G protein-coupled receptors (GPCRs), ion channels, and transporters in recent years [1], which promote the rapid development in the field of pharmacology. An emerging research strategy has utilized genetic code expansion (GCE) technology to introduce photosensitive unnatural amino acids (UAAs) into membrane proteins and synthesize photosensitivity proteins.

First, the site-directed UAA mutation technology could be traced to 1980. Like the traditional site-directed mutagenesis method, the conventional approach is to mutate a specific amino acid into any of the 20 natural amino acids, while biosynthetic UAAs with unique chemical groups break through the limitation of natural amino acid libraries. Different UAAs endow proteins with different biochemical properties, such as photosensitivity, spectral properties, and redox probes, to promote the development of new detection methods [2–8]. Generally, the amber stop codon suppression strategy is commonly used for UAA mutation, which involves mutating the target gene into a stop codon by binding the designed UAA to the suppression tRNA through the action of aminoacyl tRNA synthetase (a pair of engineered orthogonal tRNA/RS). During the protein translation process, the stop codon (usually an amber stop codon) is read through by the chemical aminoacyl tRNA so that UAAs can be inserted into the specific site of the protein. This process was later called “GCE Technology” [9].

**Figure 1:**
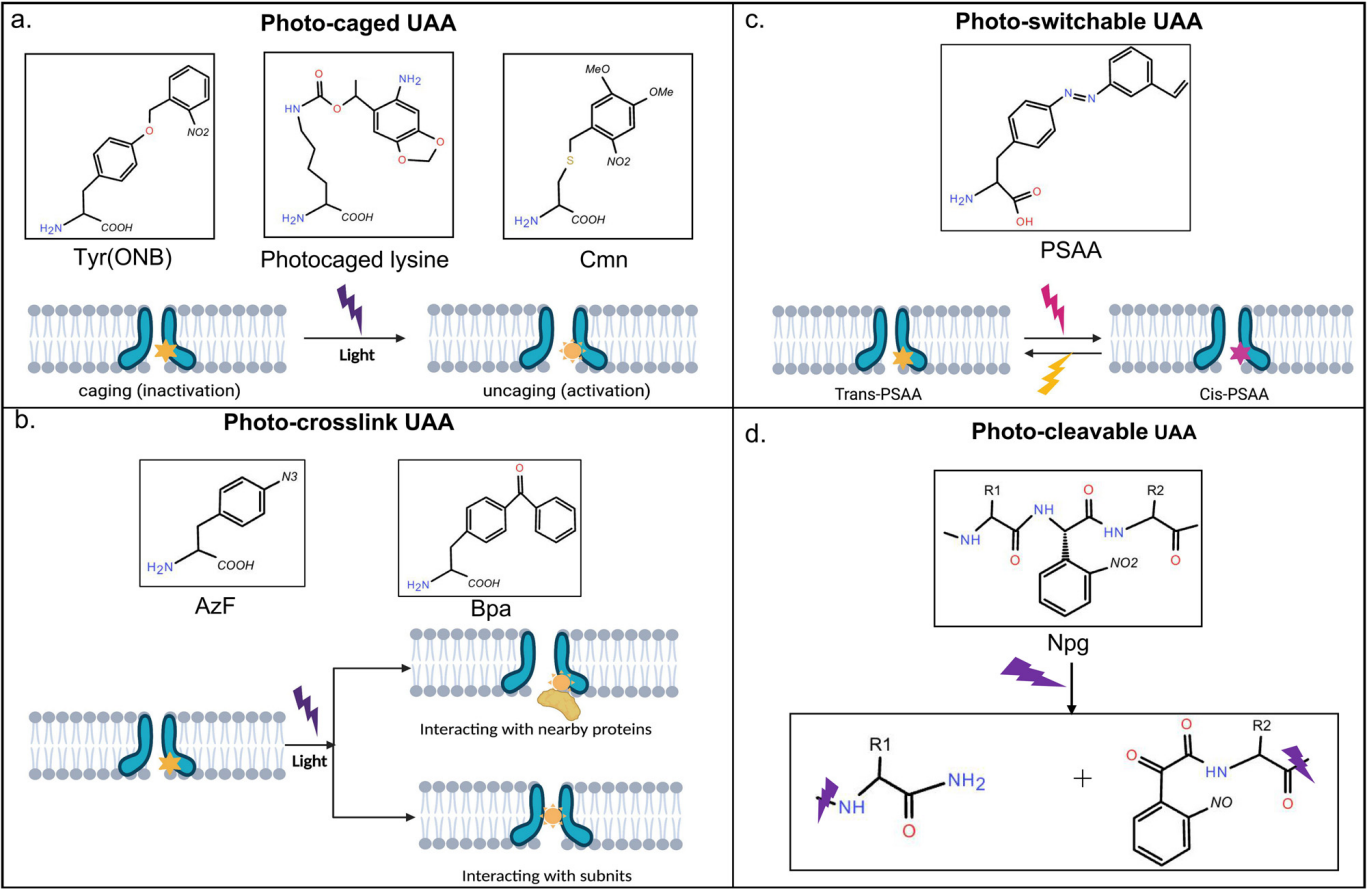
Four types of photo-sensitive UAAs. Here, we have shown the general properties of the same type UAAs when they are introduced into the specific site of the membrane protein. (a) Photo-caged UAAs. Several common photocage UAAs are shown from left to right. UAA photolysis by UV light (purple) releases the caged group and restores the activation. (b) Photo-crosslink UAAs. UV-induced photo-crosslinking to interact the target protein with the nearby protein; or photo-crosslinking UAAs introduced at key sites of the protein, physically link adjacent subunits. (c) Photo-switchable UAAs. They can rapidly turn receptor activity on or off by toggling between a cis (purple) and a trans configuration (blue), induced by two different wavelengths of light. (d) Photo-cleavable UAA. They have the characteristic to generate a “caged” protein backbone that can be cut in two upon UV illumination.

More than 100 different UAAs have been designed so far, and most of them are further developed by screening engineered aaRS/tRNA pairs for *in vivo* applications. Different UAAs containing fluorophores, post-translational modifications, metal chelators, and other novel physical and biochemical properties can bind proteins with high fidelity, efficiency, and selectivity for studying biological processes. Among them, a class of photosensitive UAAs has been shown to induce protein-specific functional changes upon light stimulation [10,11]. So far, four types of light-sensitive UAAs have been developed to make photo-insensitive proteins respond to light (Figure 1):

(1) Photo-caged UAAs: Their side chains are protected by photochemical and movable groups, which can be released from the side chains after light illumination. By encoding a “caged” UAA at the protein’s critical site, the key position’s structure will be changed. After light illumination, the “caged” protecting group will be removed to restore the protein activities. Common ones include “photo-caged” tyrosine (ONB), photo-caged lysine, caged cysteine, and caged Serine (Cmn) [12].
(2) Photo-crosslink UAAs: These UAAs contain side chain groups that respond to light. they can bind to any common functional group in nearby proteins after light exposure. Such properties make them useful in capturing protein–protein interactions, widely used in receptor–ligand interactions, protein interactions in signaling pathways, and membrane protein interactions. Many UAAs contain UV-induced crosslinking groups that are stable in organisms after photolysis, such as diazoacyl esters, aryl azide, benzophenone, and diazirine [12], which is a relatively new phenomenon for the development of photocrosslinking UAAs [10].
(3) Photo-switchable UAAs (PSAAs): These UAAs contain azobenzene groups and can switch reversibly between two protein conformations under two different wavelengths of illumination [10].
(4) Photo-cleavable UAAs: This type of UAA can be regarded as a caged backbone, which can cleave the protein backbone under optical conditions and undergo photochemical proteolysis. For example, 2-(nitrophenyl)glycine (Npg) [11] is commonly used in ion channel research.

This article introduces several application examples of using photosensitive UAAs to study typical membrane proteins in recent years. We will focus on the research of protein structure and function, downstream signal transduction, and protein–protein interaction groups, as well as summarize the application methods of some photosensitive UAAs. We will summarize the general strategy of UAAs that we have used in conjunction with our previous studies. The common strategies we summarize can be applied to other functional proteins and we highlight the broad application of photosensitive UAAs and their prospects.

## 2 Protein structure and function

**Figure 2:**
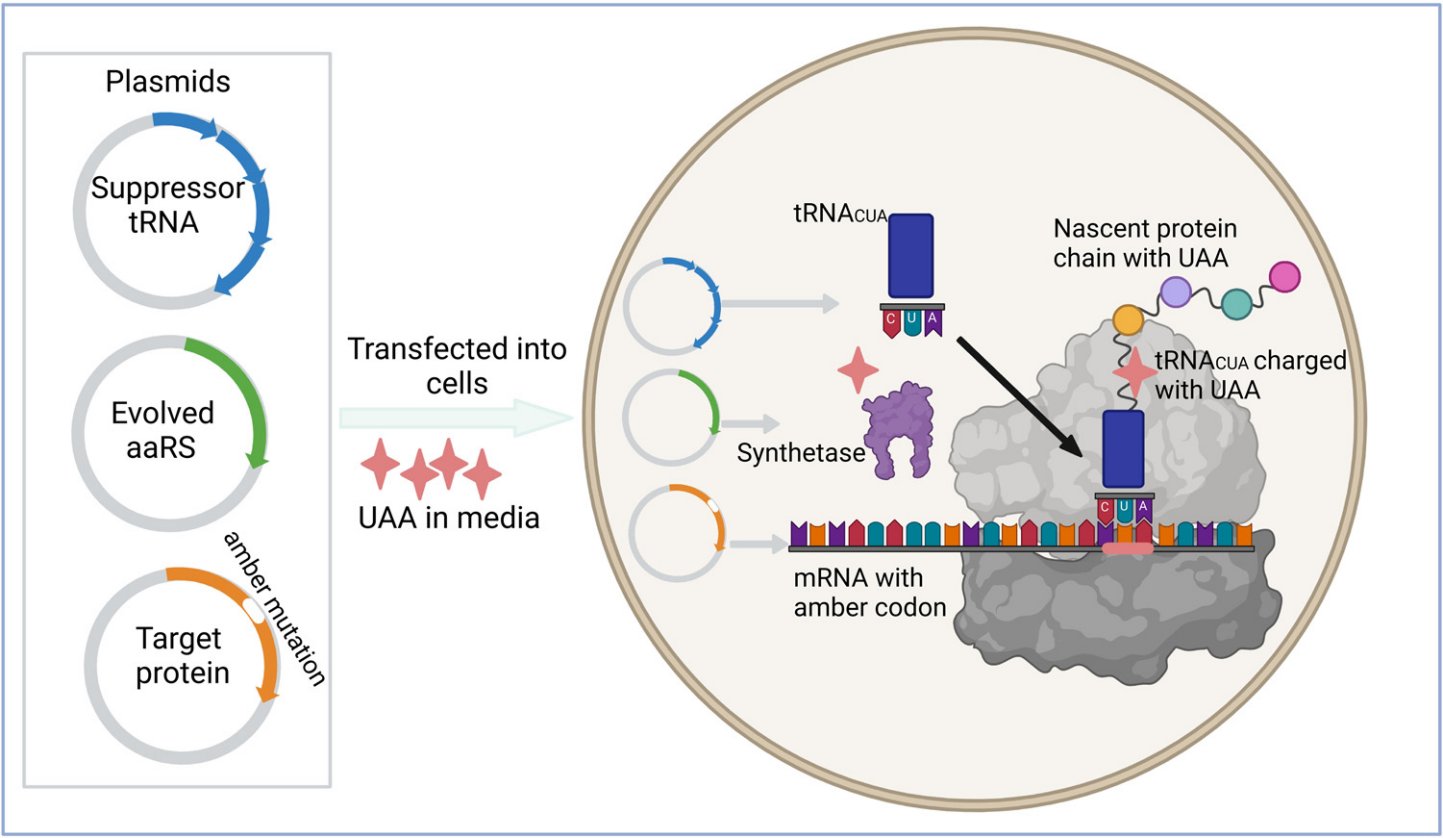
Encoding UAAs by using GCE technology in mammals. Receptor genes containing amber mutations need to be matched with appropriate orthogonal aaRS and suppression tRNA genes and then all these elements are co-transfected into cells.

Previous pioneering studies have been focused on GPCRs encoding *p*-azidophenylalanine (AzF) and *p*-benzoyl phenylalanine (*p*-Bpa). GPCR family with seven transmembrane (TM) helices is ubiquitous in eukaryotes. Usually, GPCRs are very important drug targets and can bind with hormones, cytokines, metabolites, neurotransmitters, and other signaling molecules. Through derivatized orthogonal tRNA/RS pairs, these two UAAs make full use of GCE to express photosensitive GPCRs in yeast and mammalian cells [13]. Usually, any amino acid residue can be replaced by AzF or BzF, receptor genes containing amber mutations need to be matched with appropriate orthogonal aminoacyl-tRNA synthetase (aaRS) and suppression tRNA genes, and then, all these elements are co-transfected into cells. Figure 2 shows the common method to encode UAAs by using GCE technology in mammals (Figure 2).

This photocrosslinking UAA mutation strategy was first applied to one of the GPCR members – the rhodopsin receptor. Previous studies reported the feasibility of using AzF-labeled rhodopsin receptors to track specific sites in proteins and observe their conformational changes. It was found that the most significant difference was the change in intracellular structure when the receptor reached a metastable state (MetaI state) upon light compared with the activated state [10]. AzF has infrared properties and can be widely used to study protein conformational changes. By using this labeling method (an artificial infrared probe) to track the conformational changes of GPCRs, the antisymmetric extension of the azide group in the spectral window is about 2,100 cm^−1^, which is not affected by other protein vibrations and can promote the precise identification and characterization of GPCRs. In addition, the electrostatic and polar sensitivity of azido groups provides an attractive solution for identifying changes in specific environments during protein conformational transitions [14,15]. Meanwhile, a photo-crosslinking strategy targeting BzF and AzF has also been used to identify the ligand-binding site of chemokine CCR5 (the co-receptor of human immunodeficiency virus type 1). The research on the molecular drug maraviroc (an inhibitor targeting GPCRs) has proved that these photoactivatable UAAs can be used to study the targets of drug therapy without destroying the drug’s structure, which is of great benefit to the design and development of the GPCR-based therapy [16]. For many GRCRS, the details of ligand–receptor interactions remain unclear. A recent study used AzF to identify different ligand binding modes to the angiotensin II type 1 receptor (AT1R). By labeling AT1Rs with AzF, researchers were able to delineate important contact sites in AT1R and identify key determinants of the receptor-mediated ligand domain. This strategy is beneficial to study the conformational changes caused by the ligand-binding GPCRs [17]. Besides photo-crosslink UAAs, there is a photochemical approach to the use of photo-cleavable UAAs. Studies have reported that the photolysis of Npg (a kind of photo-cleavable UAAs) can be used to cut the protein backbone of the GABA type A receptor subunit region, resulting in pharmacological changes [11].

Later studies constructed photosensitive ionotropic glutamate receptor proteins, including AMPA receptors [18] and NMDA receptors [19,20,21]. Glutamate receptors consist of tetramers of subunits and are critical for neuronal development and plasticity. They are responsible for fast excitatory synaptic transmission. It is still unclear how the conformation changes in the TM domain during the ion channel activation and desensitization process [22]. The most used photosensitive UAAs for studying ion channels are photo-crosslinking probes, especially two photo-crosslinking small molecules, AzF and Bpa (BzF or Bpa), as introduced above. Klippenstein [22] introduced Bpa at S729 or G725 site of GluA2 (a subunit of the AMPA receptor). These two sites are located at the subunit interface of the ligand-binding domain (LBD), and studies have shown that these sites allow UV-induced photoinactivation effects [23]. As research progresses, recent research explores the dynamic characteristics of the AMPA TM domain; this study selectively inserted AzF or BzF at 30 sites in the TM domain of GluA2. The photochemical characteristics of these two photosensitive UAAs allow UV light to dynamically interfere with the receptor structure at specific sites, which promotes the following research on the related drugs’ intervention [22].

The photosensitive NMDA receptor exhibited different mechanisms from that of the AMPA receptor when AzF was introduced at their subunit interfaces, respectively. The subunit interface located at the N-terminal domain is adjacent to the LBD, at which multiple binding sites for allosteric regulation have been previously identified. NMDA receptors mediate the excitatory synaptic transmission related to learning and memory, and functional NMDA receptors usually include two GluN1 and two GluN2 subunits. Tian [20] used the NMDA receptor as a model and combined photochemical methods to explore the different structural regulatory pathways. They focussed on mechanism of the allosteric inhibitor Ifenprodil bound to NMDAR’s NTD interface and the endogenous inhibitor Zn^2+^. They have identified a novel allosteric enhancement mechanism by specifically introducing photo-crosslinking small molecules at the Ifenprodil binding interface between GluN1 and GluN2 subunits [21,24]. Studies have shown that the NMDA subunits GluN1-Y109AzF/GluN2B exhibited UV-induced inactivation, while GluN1/GluN2B-F114AzF exhibited UV-induced enhancement. These two subunits exhibited different light-induced regulation effects. For GluN1-Y109AzF/GluN2B, due to the UV-induced crosslinking in the GluN1 subunit, the receptor changes from the wild-type conformation to the low channel open probability (Po) state. For GluN1/GluN2B-F114AzF, it is UV-induced low (Po) state to wild-type state [24]. These photosensitive NMDA receptors serve as novel “photoreceptors” for probing receptor function, constructing a new platform for revealing new allosteric regulatory mechanisms and, at the same time, promoting the development of subtype-selective therapeutics. This design concept – interfacial crosslinking (receptor–ligand interface) has been extended to potassium channels in recent years. These studies have emphasized the pharmacological mechanism of the receptors, making them promising drug targets for the treatment of diseases [25–28]. Furthermore, the introduction of PSAAs at multiple sites generates photo-switchable NMDA receptors, demonstrating the feasibility of reversible control of neuronal receptors under two different wavelengths of illumination. For example, when a PSAA is inserted into the pores of the conserved TM region of the NMDA receptor, the photoisomerization leads to conformational changes in the membrane protein, which then affects the gated and permeable properties of the receptor. This type of UAAs helps understand the biophysical properties of TM pore locations and reveal the role of specific side chains on different receptor properties, such as agonist sensitivity, channel opening probability, and permeability [29].

Overall, photosensitive UAAs provide a new approach to studying the functional properties of ion channels at the molecular level, such as the characteristic of structure, gating, regulation, or assembly. UAAs with photo-crosslinking can precisely locate the interaction domains of bait proteins and provide complex structural information of interacting proteins. The development of this area opens up new possibilities for the intact study of biological ion channels in complex environments.

## 3 Protein activity and signaling transduction

Light has a high degree of temporal and spatial dynamics in regulating cell signaling networks as a preferred external regulatory element. A typical example is the photochemical regulation of the kinase signals controlled by photosensitive UAAs in human cells, first achieved by “photocaged” UAAs [30]. For example, in mammalian cell systems, a “caged” lysine was directly introduced into the MEK1 kinase and replaced a highly conserved lysine essential for ATP anchoring in the kinase catalytic domain [30]. MEK is a part of the signaling network that transmits signals through ERK1/2 phosphorylation. This caged kinase is entirely inactive and loses catalytic activity, which will not be restored until the photolabile group is removed upon UV exposure, as measured by the phosphorylation levels of downstream substrates of ERK1/2. Usually, ERK activated by MEK1 is translocated from the cytoplasm to the nucleus, and in combination with fluorescent tags such as EGFP, we can precisely measure the kinetics of translocation [31,32].

**Figure 3:**
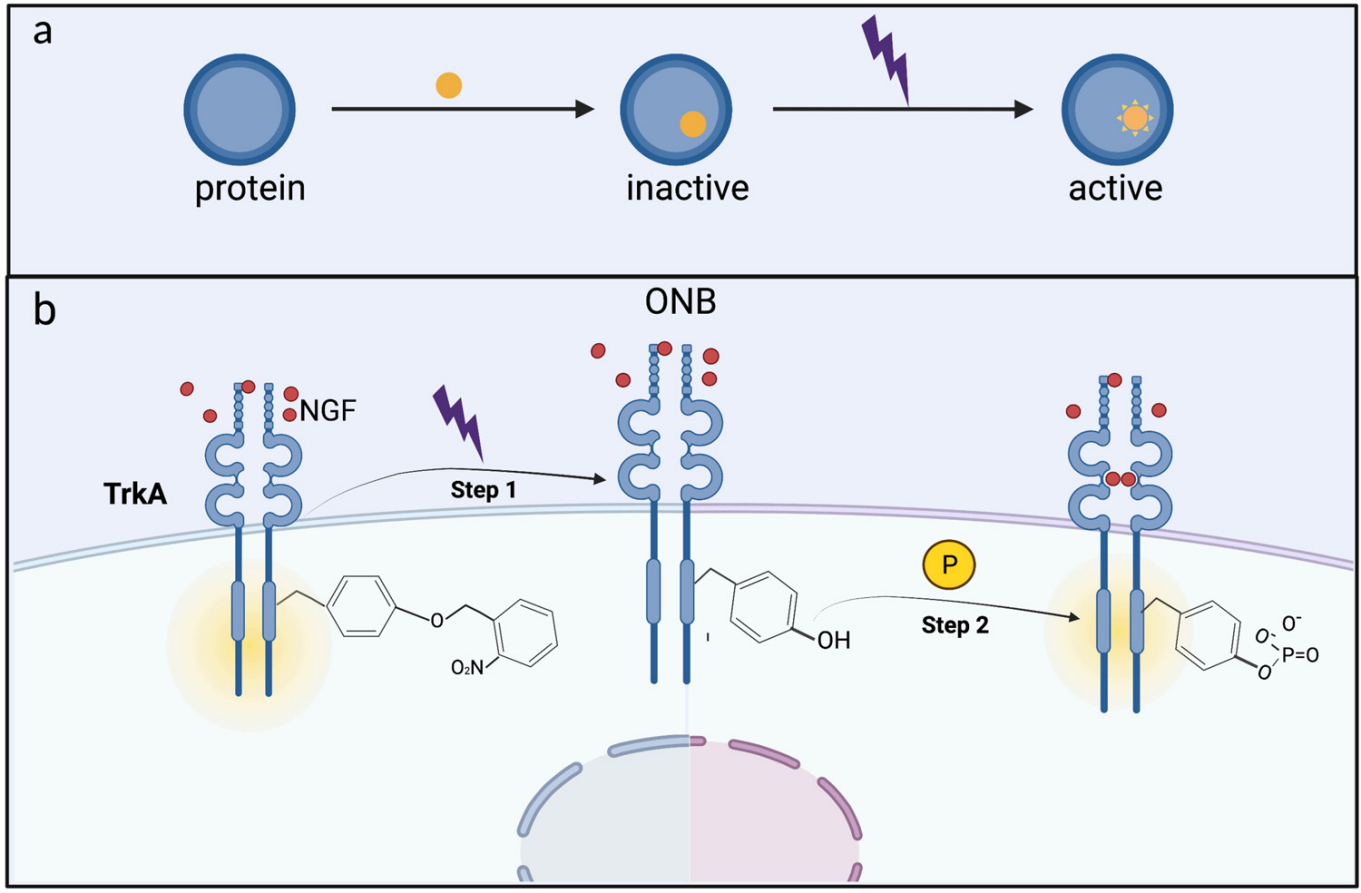
Optical activation of Trk receptor by site-specifically introducing photocaged tyrosine (ONB). (a) Inserting a light-sensitive UAA into a protein can transform a protein to respond to light. Upon light stimulation, protein changes from the ground state to the activated state due to the photo-responses of the UAA, which leads to a functional readout. (b) Three main categories of light responsive UAAs: caged amino acids, photo-cross-linking amino acids, and photo-switchable amino acids. When these UAAs are introduced into ion channels (black channels) or other proteins (grey circles), various biological responses have been observed.

Inspired by previous works, our study published in late 2020 focused on a system for optically and site-specifically controlling the membrane protein functions [32]. We installed a light-controlled “switch” on a specific site of the target protein TrkA (a member of the RTK family) to control specific signaling pathways. Two photosensitive UAAs with different photochemical reactions combined with site-directed mutagenesis (phosphorylation sites) resulted in precise light-controlled activation of receptor tyrosine kinase and provided a novel concept for a more accurate study of the TrkA phosphorylation signaling pathway [33]. We selected two photosensitive tyrosine derivatives, ONB and AzF. They are chemically structurally distinct from tyrosine, and both prevent phosphorylation before photoactivation. After UV treatment, these two different UAAs provide a unique means to control MAPK/ERK pathways by light, in which ONB releases “photocage” groups (repress tyrosine phosphorylation), thereby forming a light-dependent “gain-of-function” phenotype (Figure 3). At the same time, AzF may crosslink with neighboring molecules after light exposure, which can directly produce NGF-independent phosphorylation effects at certain sites. This strategy provides a light-controlled means to analyze the contribution of different tyrosine phosphorylation sites to specific pathway activation during cell signaling transduction, which conventional mutagenesis methods cannot.

Conventional strategies to interfere with signaling networks include gene knockout and knock in, but the drawback of these methods is that these methods require the long-term induction. In contrast, the activation of such photosensitive UAAs with different photochemical properties can be very accurate to control functional gain with spatial and temporal resolution, which satisfies various dynamic studies of cells.

## 4 Protein–protein interactome

**Figure 4:**
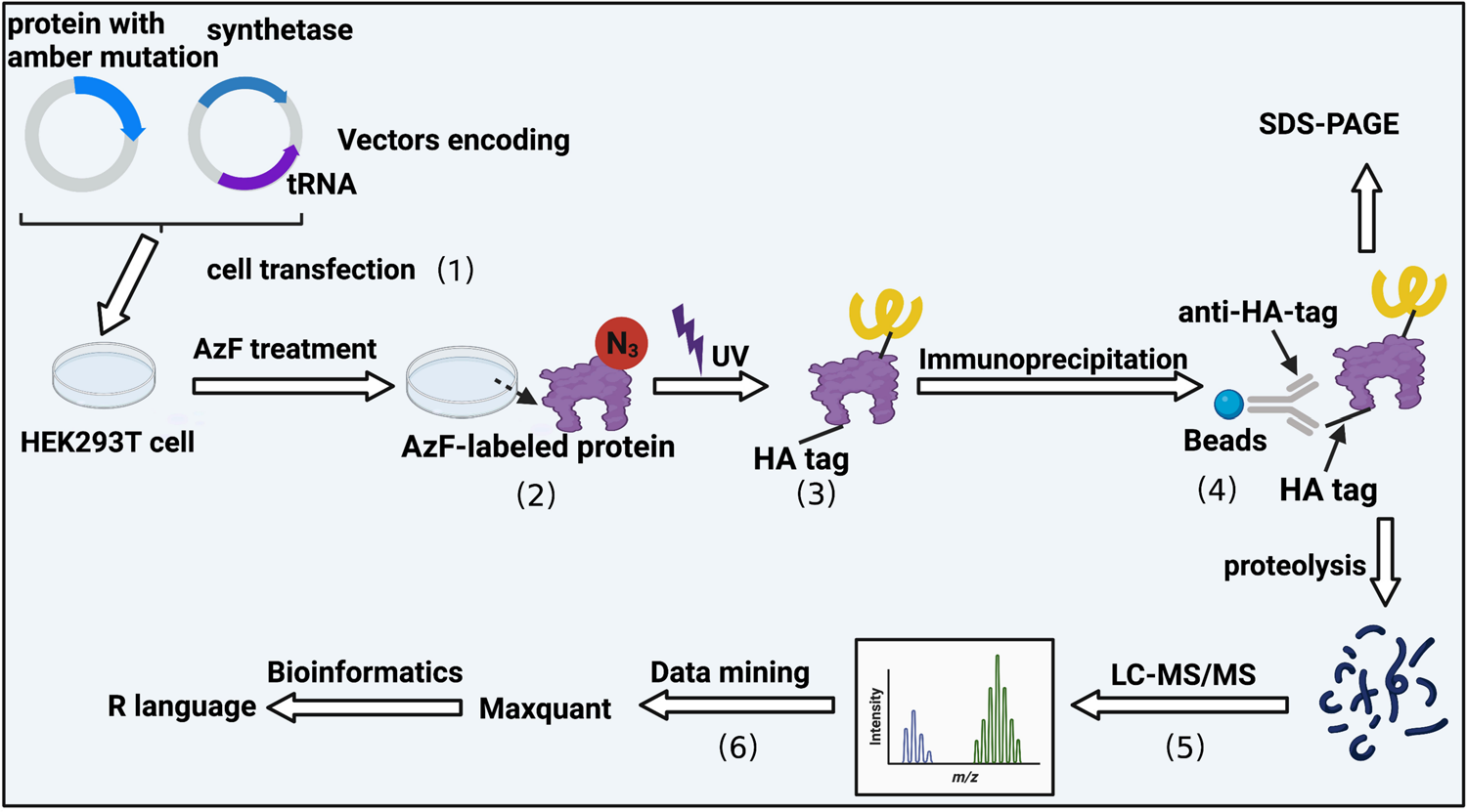
General photocrosslinking workflow for photocrosslink molecular – AzF. (1) The HA-tagged protein and the tRNA/RS pairs specifically recognizing AzF were co-transfected into HKE293T cells; (2) AzF-labeled protein; (3) the target protein covalently bound with the interacting proteins under (UV) irradiation; (4) immunopurification and HA-tagged protein enrichment; (5) separation of the target protein and its crosslinked complexes; after trypsin hydrolysis, conduct liquid chromatography–tandem mass spectrometry detection; and (6) bioinformatics statistical analysis and visual display of data.

In addition to capturing functional information, UAAs with photo-crosslinking properties, including AzF and Bpa, have been installed into membrane proteins by genetic recoding for photoaffinity crosslinking experiments [34]. The interaction of membrane proteins is crucial for many biological processes. The crosslinking properties of photosensitive UAAs can capture natural intracellular protein interactions. Our group recently reported a study of the interaction of LAT3 by using photo-crosslink UAAs. This method is based on the optical control and proteomics, a biological approach to studying cellular processes has been conceptualized as optoproteomics [10]. The amino acid transporter LAT3 is a membrane protein, and studies have reported that it is highly expressed in cancer tissues, but its interaction partners and the mechanism are still unclear. While AzF has been widely used in membrane protein interaction studies due to its highly efficient photo-crosslinking properties. Here, we describe a general photocrosslinking workflow of using AzF (Figure 4); it includes the expression of mutants, capturing and immunoprecipitation of the protein complex, and mass spectrometry identification [35]. Wang et al. used this strategy to identify transient and weak interacting partners of LAT3 in living cells [34]. The core strategy of this optoproteomics lies in the expression of UAA-mutated proteins at specific sites. The key points are the choice of cellular system and the efficiency of site-directed UAA incorporation. In addition, tags are usually assigned to exogenous proteins to exclude interference from endogenous proteins.

However, the detection of native protein interactions is currently still challenging, mainly due to the low affinity and the environment dependence [36]. By using UAAs with photocrosslinking properties, such as benzoylphenone (*p*-benzoyl-l-phenylalanine), azides (such as the *p*-azido-l-phenylalanine, AzF), or diazirines, they can effectively crosslink proteins within a distance of 3–5 Å after photoactivation, and this chemical crosslinking method has been established and used *in vivo*. Interactionism studies of several proteins in cells have been established [37,38]. The development of emerging optoproteomic strategies that satisfy both protein specificity and site specificity will have important implications for studying membrane proteins such as membrane transporters in related diseases [35].

## 5 Conclusions

We summarize the following points for this review: (1) we focus on the recent examples of light-control studies on typical membrane proteins by using genetically encoded photosensitive UAAs. This strategy was earlier applied to ion channels and membrane receptors; it is used to study its dynamic conformational changes [39], especially in the study of ion channel receptors. UAAs are effectively introduced into ion channels to make ion channel photosensitizing. These different photochemical properties of UAA can be used as biophysical probes or drivers to manipulate ion channels, providing new insights into gating properties, pharmacology, and receptor–ligand interactions. Photosensitive UAAs can be effectively incorporated into functional proteins in mammalian cells, laying the foundation for the expanded application of UAAs. They have the potential to address fundamental questions about the biology of natural receptors. Given their wide range of uses and rich functional applications, the introduction of UAAs is expected to become increasingly popular in studying protein structure and function studies [40,41,42]. (2) AzF which we have mentioned the most in this paper is a general UAA with a variety of applications, in addition to the role of crosslinking molecules, it can also function as infrared probes [14] or bioorthogonal conjugation (chemical means) [35]. Compared with the traditional fluorescent labeling method, AzF only needs to mutate a certain site of the target protein and can recognize changes within a distance of 3–6 Å through light sensing. This small molecule compound provides a unique means to identify the subtle structure changes of the protein [43]. In addition, the application of photocaged UAAs is a gain-of-function research method. A recent study has reported the use of this small-molecule-photocage strategy to control the activity of endogenous proteins in living cells [44]. By using photocaged UAAs to replace key active sites of the target protein, the function of the target protein is shut down or restored by light or not [45,46]. Providing a light-controlled switch, this method can provide spatiotemporal selective intervention for active site studies of endogenous proteins (not just membrane proteins) and has broad application prospects.
